# Severity of COVID-19 cases in the months of predominance of the Alpha and Delta variants

**DOI:** 10.1038/s41598-022-19125-4

**Published:** 2022-09-14

**Authors:** D. Florensa, J. Mateo, R. Spaimoc, C. Miret, S. Godoy, F. Solsona, P. Godoy

**Affiliations:** 1 Department of Computer Science, University of Lleida, Jaume II 69, 25001 Lleida, Spain; 2 Population Cancer Registry, Santa Maria University Hospital, Av. Alcalde Rovira Roure 44, 25198 Lleida, Spain; 3 Field Epidemiology, Lleida Biomedical Research Institute (IRB Lleida), Av. Alcalde Rovira Roure 80, 25198 Lleida, Spain; 4 Epidemiology Service, Department of Health, Av. Alcalde Rovira Roure 2, 25006 Lleida, Spain; 5 CIBER Epidemiology and Public Health (CIBERESP), Health Institute Carlos III, Madrid, Spain

**Keywords:** Health care, Risk factors

## Abstract

New SARS-CoV-2 may pose problems in controlling the COVID-19 pandemic for public health. We aimed to assess and compare the symptoms and severity of cases due to the Alpha and Delta variant dominance periods, taking into account the effect of COVID-19 vaccination. A prospective epidemiological study of SARS-CoV-2 in Lleida was made to determine differences between Alpha and Delta variants periods. We assessed symptoms, specific comorbidities, sociodemographic information and vaccination status. Bivariate and logistic regression analyses were used to estimate the adjusted odds ratio (aOR) and 95% confidence intervals (CI) to investigate the relationship between symptoms and severity and the variants. Alpha variant period compared to the Delta showed an increased risk of ICU admission (aOR 2.0; 95% CI 1.2–2.3) and death (aOR 2.6; 95% CI 1.8–3.9) and cases were associated with people aged > 85 years (aOR 2.1; 95% CI 1.7–2.6) and partially vaccinated (aOR 5.6; 95% CI 3.2–9.9) and unvaccinated people (aOR 27.8; 95% CI 19.7–40.5). Fever, cough and vomiting were significantly associated with the Alpha variant compared to the Delta (aOR 1.6 (95% CI 1.5–1.7), 2.0 (95% CI 1.9–2.2) and 2.5 (95% CI 2.2–2.9, respectively). Our results show that the severity and profile of clinical symptoms varied according to the variant. The risk of ICU admission and death was higher in the Alpha period compared to the Delta as it affected the elderly and cases were less vaccinated.

## Introduction

The emergence of new variants of SARS-CoV-2 has supposed a problem in controlling the COVID-19 pandemic. The COVID-19 variant of concern, B.1.1.7, colloquially known as Alpha, was first identified in the United Kingdom^1^. Then a new variant was discovered in India. The variant of concern B.1.617.2 was called Delta^2^.

Studies have analyzed the main symptoms of the Alpha variant by patient characteristics and the role of vaccination^3^. Some studies^4,5^ found an association between the Alpha variant and an increase in hospitalization, ICU admission and mortality. However, Veneti et al*.*^6^ found no differences in the risk of hospitalization between reported cases of the SARS-CoV-2 Delta and Alpha variants in Norway. Burki^7^ concluded that the Delta variant was almost twice as likely to lead to hospitalization as infection with the Alpha variant. Therefore, some studies have come to different conclusions.

Vaccination has had an important impact on virus transmission. Studies^8,9^ have shown the effectiveness of the COVID-19 vaccine in preventing severe cases due to SARS-CoV-2 and concluded that effectiveness may decrease after 6 months of follow-up even though the vaccine remains highly efficacious in preventing serious cases of COVID-19^10^. Harder et al*.*^11^ compared the effectiveness of the vaccine between the Alpha and Delta variants and found that the strength of the vaccine was reduced by 10–20%, but maintained full effectiveness against severe COVID-19. In Spain, the first doses of the vaccines were administrated at the beginning of January 2021. The ≥ 85 and 75–84 years age groups were the first to receive the vaccines^12^.

Studies of the Alpha and Delta variants have found differing outcomes. However, they mostly concluded that the Alpha variant increased the risk of severe cases, although some studies resulted in different conclusions. Our study added and analyzed all symptoms seen in recorded patients during the dominance of each variant and their vaccination status.

The aim of this study was to assess and compare the symptoms and severity of COVID-19 cases in the months when the Alpha variant predominated, taking into account the effect of COVID-19 vaccination. We also compared the differing patient profiles and comorbidities between the Alpha and Delta periods.

## Methods

We made a prospective epidemiological study of SARS-CoV-2 in the province of Lleida (Spain) to determine differences between the periods of predominance of the Alpha and Delta variants. We analyzed symptoms, specific comorbidities, sociodemographic information and the COVID-19 vaccination status. The main sources of information were recorded cases from the Lleida and Alt Pirineu-Aran epidemiological service, which is part of the Catalan Department of Health. SARS-CoV-2 cases were detected by PCR (polymerase chain reaction) and antigen tests. Cases were validated, studied and controlled by epidemiological professionals in order to monitor the disease and observe the evolution of the pandemic in the two outbreaks.

### Data

The data collected correspond to new cases from two consecutive waves of the pandemic with circulation of the Alpha and Delta variants. As we did not know the genome sequence for all confirmed cases, each wave was characterized, at baseline, by a prevalence of 50% of the sequenced genome for the respective variant (Alpha or Delta) and, subsequently by a prevalence of > 90% during peak hospital admissions. Data were collected by Department of Health public health officials by telephone interview with each patient and checking and/or reviewing clinical records, as is normal for data collection by the public epidemiological surveillance system. The first period comprises records from January 1, 2021 to March 31, 2021, with circulation of the Alpha variant in the Lleida region. The second period, with circulation of the Delta variant, comprises records from April 1, 2021 to July 31, 2021^11^. In the second period, the government permitted mobility, social contacts and the opening of night clubs. The study variables were variant dominance period (Alpha, Delta); age group (0–14, 15–34, 35–44, 45–54, 55–64, 65–74, 75–84, ≥ 85 years); sex (male, female); asymptomatic; hospitalized; ICU; death; fever; cough; pneumonia; sore throat; shivers; dyspnea; vomiting; diarrhea; respiratory distress syndrome; acute renal failure; cardiovascular disease; neurological disease; vaccinated (fully, half, non). Patients were considered fully vaccinated only if two vaccine doses were received and the time between the second vaccination and the onset of COVID-19 was > 14 days. Patients were considered partially vaccinated if only one effective vaccine dose was received. Patients were considered unvaccinated if no vaccine had been received or the first vaccination was given < 14 days before the onset of COVID-19.

### Statistics

We compared SARS-CoV-2 cases during the predominance of the Alpha variant with the predominance of the Delta variant. We compared sociodemographic information, symptoms, risk factors of the patients and the effect of vaccination. In the first analysis, crude odds ratios (OR) were used to compare the differences in positive cases between the variants.

A bivariate analysis was made to investigate relationships between the dependent variable (the Alpha or Delta variants), and independent variables (UCI, death, sociodemographic data, symptoms, risk factors and vaccination status).

The patient profile of the Alpha variant compared with the Delta variant as a reference category was analyzed using logistic regression to estimate probabilities, OR and their corresponding 95% confidence intervals (CI).

The logistic regression analysis was based on forward selection. This involves starting with no variables in the model, adding the variable (if any) whose inclusion gives the most significant improvement to the fit, and repeating this process until none improve the model.

### Ethics statement

All data were anonymized to protect patient privacy and confidentiality. This study was part of the public health response to coronavirus outbreaks. This project was approved by the reference ethics committee (Committee of Ethics and Clinical Research of Lleida—CEIC). The patients were interviewed by telephone to obtain the COVID-19 symptoms and vaccine information. They were informed about this study in this interview, and they provided informed consent during enrolment. All methods were carried out in accordance with relevant guidelines and regulations.

## Results

**Figure 1:**
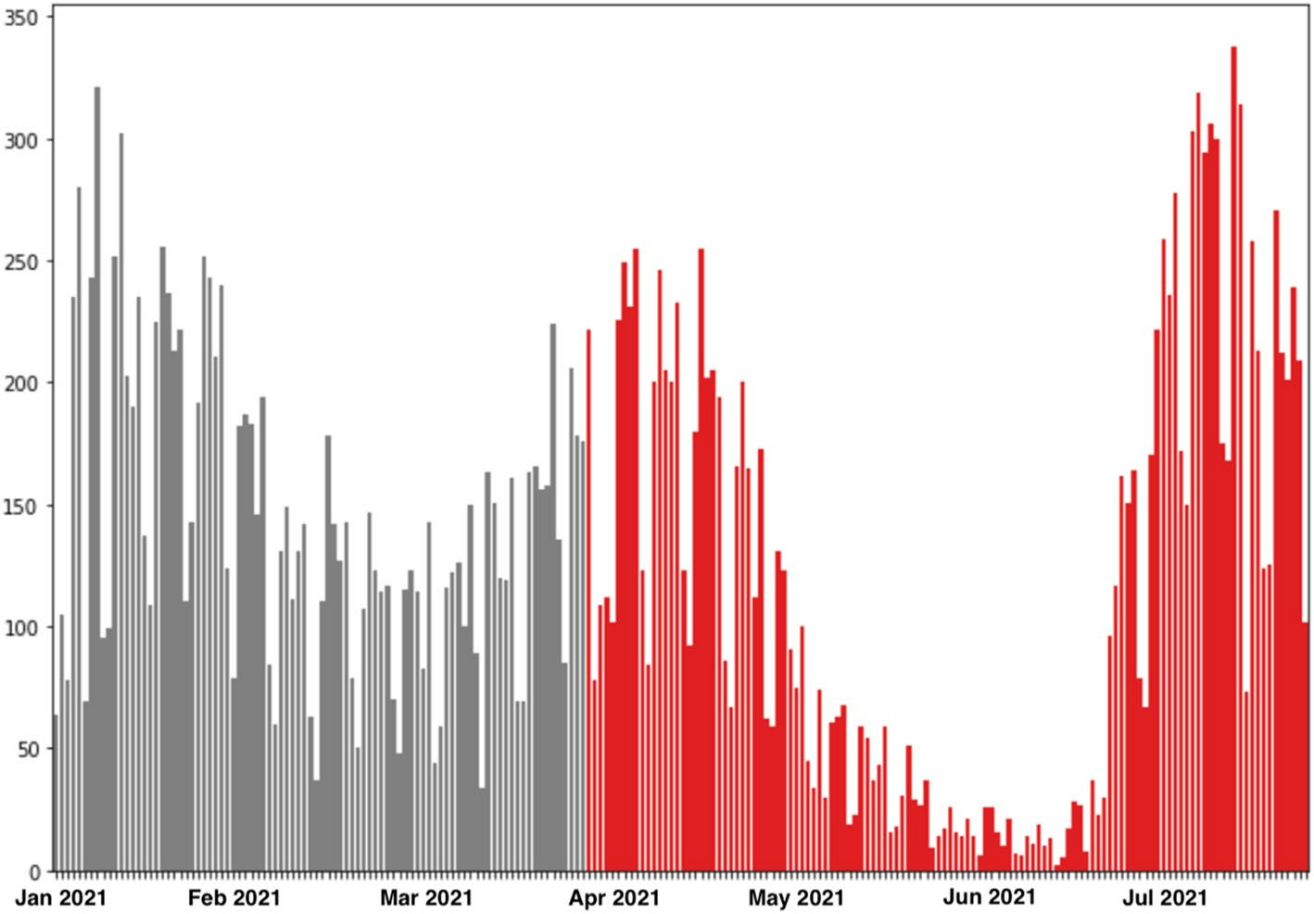
Epidemiologic curve from 01-001-2021 until 31-07-2021. Grey shows the predominance of the Alpha variant and red the predominance of the Delta variant.

The overall data set consisted of 27,011 SARS-CoV-2 cases confirmed by PCR and antigen tests. When the Alpha variant was predominant, 13,037 cases were detected, and when the Delta variant predominated 13,974 cases were detected (Fig. 1).

### Bivariate analysis

Patient profiles, symptoms, comorbidities and the vaccination effect between the Delta and Alpha variants were analyzed and compared (Table 1). During the Alpha period, 6645 (51%) women were recorded compared with 6727 (48.1%) in the Delta period (P < 0.001). There were differences between age groups: during the Alpha period there were 2948 (23%) cases in the 15–34 age group compared with 5308 (38%) during the Delta period. However, in the 55–64 years and older age groups, the number of cases of the Delta variant decreased compared with the Alpha variant (P < 0.001).

**Table 1:** Bivariate analysis of COVID-19 cases between the predominance of the Alpha and Delta variants in Lleida region (Spain).

	Total		Alpha		Delta		Crude OR	95% CI	P-value
	Count	%	Count	%	Count	%			
**Sex**									
Female	13,372	49.5	6645	51	6727	48.1	Ref. group	–	–
Male	13,639	50.5	6392	49	7247	51.9	0.9	0.8–0.9	< 0.001
**Age**									
[0–14)	4450	16.5	2256	17.0	2194	15.7	Ref. group	–	
[15–34)	8256	30.6	2948	23.0	5308	38.0	0.5	0.5–0.6	< 0.001
[35–44)	3975	14.7	1970	15.0	2005	14.3	1.0	0.9–1.0	0.2
[45–54)	3841	14.2	2076	16.0	1765	12.6	1.1	1.0–1.2	0.001
[55–64)	2929	10.8	1647	13.0	1282	9.2	1.3	1.1–1.4	< 0.001
[65–74)	1742	6.4	963	7.4	779	5.6	1.2	1.0–1.3	< 0.001
[75–84)	1063	3.9	646	5	417	3	1.5	1.3–1.7	< 0.001
[85–)	755	2.8	531	4.1	224	1.6	2.3	1.9–2.7	< 0.001
Hospitalized*	736	2.7	491	3.8	245	1.8	2.2	1.8–2.6	< 0.001
ICU*	81	0.3	56	0.4	25	0.2	2.4	1.5–3.9	< 0.001
Death*	197	0.7	161	1.2	36	0.3	4.8	3.4–7.0	< 0.001
Symptoms*									
Fever	5614	20.8	4511	35	1103	7.9	6.2	5.7–6.6	< 0.001
Cough	5640	20.9	4626	36	1014	7.3	7.0	6.5–7.6	< 0.001
Pneumonia	32	0.1	25	0.2	7	0.1	3.8	1.7–8.9	< 0.001
Sore throat	3665	13.6	2232	17	1433	10.3	1.8	1.7–1.9	< 0.001
Shivers	3259	12.1	2049	16	1210	8.7	2.0	1.8–2.1	< 0.001
Dyspnea	1104	4.1	745	5.7	359	2.6	2.3	2.0–2.6	< 0.001
Vomits	2119	7.8	1604	12	515	3.7	3.7	3.3–4.1	< 0.001
Diarrhea	2684	9.9	1767	14	917	6.6	2.2	2.1–2.4	< 0.001
Comorbidities*									
RDS	50	0.2	42	0.3	8	0.1	5.6	2.6–12.0	< 0.001
Acute renal failure	39	0.1	34	0.3	5	0	7.3	2.9–18.7	< 0.001
Cardiovascular dis	581	2.2	401	3.1	180	1.3	2.4	2.0–2.9	< 0.001
Neurological dis	226	0.8	171	1.3	55	0.4	3.3	2.5–4.6	< 0.001
**Vaccinated**									
Fully	796	2.9	26	0.2	770	5.5	Ref. group	–	–
Half	172	0.6	29	0.2	143	1	6.0	3.4–10.5	< 0.001
None	26,043	96.5	12,982	99.6	13,061	93.5	29.4	19.9–43.5	< 0.001

*OR* Odd Ratio, *Ref. group* Reference Group, *CI* Confidence Interval, *ICU* Intensive Care Unit, *RDS* Respiratory Distress Symptom, *Cardiovascular dis* cardiovascular diseases, *Neurological dis* neurological diseases.

*For the variables *Hospitalized, ICU, Death*, and for *Symptoms* and *Comorbidities* the reference group was no.

Hospitalized cases were higher during the Alpha period (n = 491; 3.8%) than in the Delta period (n = 245; 1.8%) and 56 (0.4%) cases required ICU admission during the Alpha period and 25 (0.2%) during the Delta period (P < 0.001). Death was also significant with 161 (1.2%) cases related to the Alpha variant and 36 (0.3%) to the Delta variant (P < 0.001).

Regarding symptoms, the P-value was significant for all symptoms (P < 0.001). Major differences between variants were observed in the number of patients with fever and cough. There were also significant differences related to comorbidities. Vaccination also was significant (P < 0.001) in all categories. The number of fully-vaccinated positive cases increased notably in the Delta period.

### Logistic regression model

Sociodemographic information, symptoms and comorbidities differed widely (Table 2). Male sex was significant during the Alpha period (aOR 0.9; 95% CI 0.9–1.0). The 15–34 years age group showed the greatest significance [15–34] (aOR 0.5; 95% CI 0.4–0.5). The 35–44 years age group was also significant (aOR 0.7; 95% CI 0.7–0.8). There were significant differences in the elderly (aOR 2.7; 95% CI 2.2–3.2). Differences in hospitalization were not significant, but the risk of ICU admission was higher during the Alpha variant period (aOR 1.7; 95% CI 1.1–2.7) and the aOR for death was also significant (P = 2.7, 95% CI 1.9–4.0).

**Table 2:** Logistic regression analysis of COVID-19 cases between the predominance of Alpha and Delta variants in Lleida region (Spain).

	Adjusted odds ratio	95% CI	P-value
Gender female	1.0	Ref. group	–
Gender male	0.9	0.8–0.9	0.001
Age [0–14)	1.0	Ref. group	–
Age [15–34)	0.4	0.4–0.5	< 0.001
Age [35–44)	0.7	0.6–0.8	< 0.001
Age [45–54)	0.9	0.8–0.9	0.007
Age [55–64)	0.9	0.8–1.0	0.2
Age [65–74)	0.8	0.7–0.9	0.04
Age [75–84)	1.2	1.0–1.4	0.02
Age [85–)	2.7	2.2–3.2	< 0.001
Hospitalized	1.0	0.8–1.1	0.7
ICU	1.7	1.1–2.7	0.04
Death	2.7	1.9–4.0	< 0.001
Fever	3.7	3.4–4.0	< 0.001
Cough	4.3	4.0–4.6	< 0.001
Pneumonia	0.8	0.3–1.6	0.5
Sore throat	1.2	1.1–1.2	< 0.001
Shivers	0.9	0.8–1.0	0.1
Dyspnea	1.1	1.0–1.2	0.2
Vomits	2.7	2.4–3.2	< 0.001
Diarrhea	0.3	0.3–0.4	< 0.001
Respiratory distress syndrome	3.0	1.6–6.3	0.001
Acute renal failure	2.9	1.4–7.3	0.03
Cardiovascular disease	1.3	1.1–1.5	0.01
Fully-vaccinated	1.0	Ref. group	–
Half-vaccinated	5.6	3.2–9.2	< 0.001
None-vaccinated	35.7	25.7–51.3	< 0.001

In the logistic regression analysis, the reference category of the dependent variable (Alpha or Delta variant period) was the Delta variant period.

*CI* Confidence Intervals, *Ref. group* Reference Group, *ICU* Intensive Care Unit, *RDS* Respiratory Distress Symptom.

Fever and cough showed significant results with aORs of 3.7 (95% CI 3.4–4.0) and 4.3 (95% CI 4.0–4.6). Sore throat was also significant with a higher risk with the Alpha variant (aOR 1.2, 95% CI 1.1–1.2). Digestive symptoms were also. The OR for vomiting was 2.7 (95% CI 2.4–3.2) and for diarrhea it was 0.6 (95% CI: 0.5–0.7). Acute renal failure was also significant (aOR 2.9, 95% CI 1.4–7.3).

Respiratory and cardiovascular diseases were significant (aOR 3.0; 95% CI 1.7–6.3 and aOR 1.3; 95% CI 1.1–1.5, respectively). Finally, vaccination also produced significant results. The aOR for partially-vaccinated cases was 5.6 (95% CI 3.2–9.2) and for non-vaccinated cases it was 35.7 (95% CI 25.7–51.3).

## Discussion

The risk of ICU admission and death was higher during the Alpha period compared to the Delta period after adjustment in the regression model for the importance effect of age and partial vaccination or lack of vaccination. In addition, in cases in Alpha period with respect to the Delta, there were some clinical differences, such as a higher percentage of fever, cough and vomiting.

This study was based on a previous one^13^, which described reported cases and identified disease severity risk factors in the first wave in Spain but did not compare this with waves caused by other variants. A later study in Navarra found that the emergence of the Delta variant in Spain was associated with an increased proportion of cases in young people at a time when increased vaccination coverage led to increases in mobility and social interaction, and the relaxation of some preventive measures^14^. Another early study analyzed the transmissibility and global spread of COVID-19 variants and their differences in terms of effects. Twohig et al.^15^ analyzed the risk of hospital admission risk from the Delta and Alpha variants and found higher hospital admission and emergency care risks in unvaccinated patients during the Delta period. mRNA vaccines were shown to significantly reduce the risk of hospitalization and death. Bernal et al*.*^16^ analyzed the effectiveness of COVID-19 vaccines against the Delta variant and suggested a lower degree of protection than against the Alpha variant, even though they still guaranteed a high level of effectiveness.

Studies have analyzed and compared the symptoms and effectiveness of the vaccine against the COVID-19 variants, with differing results. Some studies found that the Alpha variant may increase the risk of ICU admission and mortality^4,5^, while others concluded there were no significant differences^6^. However, after reviewing earlier literature about the effectiveness of vaccines and the new variants, most studies have suggested that vaccine protection was higher against the Alpha variant but that their effectiveness in preventing severe cases caused by the Delta variant remained high.

One of the most significant results was in the 15–34 years age group^17^, in which the risk from the Delta variant was higher. Similar results were seen in the 35–44 and 65–74-years age groups. The latter group started to receive the first vaccine doses just when the Delta variant started to become predominant^18^. Therefore, they were still exposed to the virus. However, older people, specifically the ≥ 85 years age group, had a higher risk from the Alpha variant than from the Delta variant because, in the earlier period they had not received two doses of the vaccine^19^. During the wave caused by the Delta variant, these groups were fully vaccinated.

The number of cases requiring ICU admission, and mortality, during the Alpha period was higher. The logistic regression analysis confirmed the higher risk from the Alpha variant. The risk of ICU admission due to the Alpha variant was double the risk in the Delta period^20,21^. Mortality was also higher in the Alpha period, perhaps because the Alpha variant was more aggressive^4,5^.

The most significant symptoms observed during the Alpha period were fever and cough, which may indicate a higher degree of severity^22^. The incidence of vomiting was also higher with the Alpha variant, but this may not be related to the risk of hospitalization, ICU admission or death^23^. These differences suggest that symptoms could vary depending on the variant given that the genetic composition of the COVID-19 variants differs^24^.

Outcomes in partially and unvaccinated patients were also significant compared with those fully-vaccinated. Partial vaccination signified a higher risk compared with full vaccination during the Alpha period, as did non-vaccination. The risk in unvaccinated patients was significantly higher than for the fully-vaccinated. These outcomes suggest a high effectiveness of current vaccines against severity and death^25^.

This study has some limitations. The transition between the predominance of the Alpha and Delta variants may mean some cases are included in the wrong period. Some asymptomatic patients may have developed symptoms after the interview and not been registered. Some symptoms could also have appeared after the interview and not been recorded. Likewise, the COVID history could not be taken into account due to difficulties in accessing this information. The logistic regression model took into account the main variables involved, such as vaccination and morbidity, but there could still be a residual confounding effect in the results.

In conclusion, our results suggest that more severe cases appeared during the predominance of the Alpha variant. This may show that the Alpha variant is more aggressive than the Delta, depending on the case. The risk of ICU admission, and deaths, were higher in the Alpha period, as were fever, cough, and vomiting. The lack of vaccination was the main risk factor in people infected by the Alpha variant, suggesting vaccines are effective in avoiding UCI admission and death.

These results show further study of vaccine effectiveness against new variants are necessary and the importance of vaccines in maintaining high levels of protection.

## Data Availability

The dataset is available from the corresponding author upon reasonable request.
